# The Importance of the FUT2 rs602662 Polymorphism in the Risk of Cardiovascular Complications in Patients after Kidney Transplantation

**DOI:** 10.3390/ijms25126562

**Published:** 2024-06-14

**Authors:** Maciej Józef Kotowski, Piotr Ostrowski, Jerzy Sieńko, Bogusław Czerny, Karol Tejchman, Bogusław Machaliński, Aleksandra Górska, Aleksandra E. Mrozikiewicz, Anna Bogacz

**Affiliations:** 1 Department of General Surgery and Transplantology, Pomeranian Medical University in Szczecin, 70-111 Szczecin, Poland; maciej.j.kotowski@gmail.com (M.J.K.); piotr.ostrowski1997@gmail.com (P.O.); ktej78@pum.edu.pl (K.T.); 2 Institute of Physical Culture Sciences, University of Szczecin, 70-453 Szczecin, Poland; jsien@poczta.onet.pl; 3 Department of Stem Cells and Regenerative Medicine, Institute of Natural Fibres and Medicinal Plants, Kolejowa 2, 62-064 Plewiska, Poland; boguslaw.czerny@iwnirz.pl (B.C.); aleksandra.gorska@iwnirz.pl (A.G.); 4 Department of Pharmacology and Pharmacoeconomics, Pomeranian Medical University in Szczecin, Żołnierska 48, 71-230 Szczecin, Poland; 5 Department of General Pathology, Pomeranian Medical University in Szczecin, 70-111 Szczecin, Poland; boguslaw.machalinski@pum.edu.pl; 6 Department of Obstetrics and Women’s Diseases, Poznan University of Medical Sciences, Polna 33, 60-535 Poznan, Poland; a.mrozikiewicz@gmail.com; 7 Department of Personalized Medicine and Cell Therapy, Regional Blood Center, Marcelińska 44, 60-354 Poznan, Poland

**Keywords:** kidney transplantation, polymorphism, immunosuppression, cardiovascular diseases, organ rejection

## Abstract

The FUT2 gene encodes an enzyme called α-1,2-fucosyltransferase, which is involved in the formation of blood group antigens AB0(H) and is also involved in the processes of vitamin B12 absorption and its transport between cells. FUT2 gene polymorphisms are associated with vitamin B12 levels in the body. Vitamin B12 deficiency associated with hyperhomocysteinemia is a major risk factor for cardiovascular diseases (CVDs), which are one of the main causes of death in patients after kidney transplantation. The aim of our study was to determine the impact of the rs602662 (G>A) polymorphism of the FUT2 gene on the functionality of transplanted kidneys and the risk of CVD in patients after kidney transplantation. The study included 402 patients treated with immunosuppression (183 patients taking cyclosporine (CsA) and 219 patients taking tacrolimus (TAC)). The analysis of the FUT2 rs602662 (G>A) polymorphism was performed using real-time PCR. Patients with CsA were more likely to be underweight (1.64% vs. 0.91%) and obese (27.87% vs. 15.98%), while those taking TAC were more likely to be of normal weight (39.27%) or overweight (43.84%). No statistically significant differences were observed comparing the mean blood pressure, both systolic and diastolic. The renal profile showed a higher median urea nitrogen concentration in patients with CsA (26.45 mg/dL (20.60–35.40) vs. 22.95 mg/dL (17.60–33.30), *p* = 0.004). The observed frequency of rs602662 alleles of the FUT2 gene was similar in the analyzed groups. The A allele was present in 43.7% of patients with CsA and 41.1% of those taking TAC (OR = 0.898; 95% CI: 0.678–1.189; *p* = 0.453). In the group with CsA, the GG genotype was present in 32.2% of patients, the GA in 48.1% and the AA in 19.7%. A similar distribution was obtained in the TAC group: GG—33.8%, GA—50.2%, and AA—16.0%. An association of genotypes containing the G allele with a higher incidence of hypertension was observed. The G allele was present in 65% of people with hypertension and in 56% of patients with normal blood pressure (*p* = 0.036). Moreover, the evaluation of the renal parameters showed no effect of the FUT2 polymorphism on the risk of organ rejection because the levels of creatinine, eGFR, potassium, and urea nitrogen were prognostic of successful transplantation. Our results suggest that the rs6022662 FUT2 polymorphism may influence the risk of cardiovascular diseases.

## 1. Introduction

Cardiovascular disease (CVD) is the leading cause of death after kidney transplantation, and its incidence is much higher than in the general population. Conventional risk factors such as diabetes, hypertension, and dyslipidemia increase the risk of CVD after transplantation. Additionally, other factors specific to organ transplantation, such as genetic polymorphisms, elevated levels of homocysteine and mycoinflammatory markers, systemic inflammation, infections, and immunosuppressive drugs play a role in CVD risk [1,2,3,4].

In case of disorders in homocysteine metabolism, its excess accumulates in the blood, the high concentration of which is a risk factor for coronary heart disease, heart attack, and stroke. A high homocysteine level, combined with a deficiency of folate and vitamin B12, causes damage to the endothelium of blood vessels. This leads to an increased risk of developing atherosclerotic lesions (the intensification of inflammation, reconstruction of vessel walls, adhesion of platelets). The role of homocysteine in the activation of blood coagulation factors V and XII and the inactivation of factors VI and VIII is also important. This effect of homocysteine on coagulation factors indicates its prothrombotic effect. Elevated levels of homocysteine in the blood may lead to peripheral vascular thrombosis, which is independent of hypercholesterolemia, hypertension, or diabetes [3,4].

The *FUT2* gene encodes an enzyme called α-1,2-fucosyltransferase, which is involved in the formation of blood group antigens AB0(H) that are located on erythrocytes, but they are also in a soluble form in body fluids (sweat, saliva, and intestinal mucus) [5,6]. Polymorphisms that reduce the activity of the α-1,2-fucosyltransferase enzyme, such as W143X in Caucasians (rs601338G>A) and I129F in Asians, cause a “non-secretory” phenotype characterized by the absence of these antigens in body secretions and consequently affect susceptibility to many pathogens and diseases [7]. Individuals who have a functional enzyme (genotype GG and GA for rs601338G>A) are called secretors, while those who lack a functional enzyme are called non-secretors (genotype AA). Non-secretory individuals have been shown to have a lower risk of childhood diarrhea and ear infections [8,9] and an increased risk of certain autoimmune diseases, including type 1 diabetes [10], psoriasis [11,12] and inflammatory bowel disease [13,14].

Moreover, it has been shown that the fructosyltransferase 2 (FUT2) is also involved in the processes of vitamin B12 absorption and its transport between cells. *FUT2* gene polymorphisms are associated with vitamin B12 levels in the body [15,16,17]. Variants of this gene have been linked to low blood levels of vitamin B12, especially when following a vegetarian diet. Vitamin B12 deficiency is also associated with a diet low in meat and dairy products, total or partial resection of the stomach, resection of the ileum, the use of metformin, drugs blocking the secretion of gastric acid, and others [18]. Vitamin B12 deficiency leads to hematological and neurological symptoms, including glossitis, fatigue, macrocytic anemia, and peripheral neuropathy. Additionally, vitamin B12 deficiency is strongly associated with hyperhomocysteinemia, which is a major risk factor for cardiovascular diseases (CVDs) [18].

In kidney transplant recipients, special attention is paid to avoiding the risk of side effects and comorbidities that may lead to transplant failure or death, in particular obesity, infections, cancer, and cardiovascular diseases [19,20,21]. Diarrhea is a common symptom in kidney transplant recipients and may be related to the use of immunosuppressive drugs [22,23]. Among the immunosuppressive drugs, mycophenolate mofetil is extensively hydrolyzed to mycophenolic acid by esterases in the stomach, small intestine, blood, liver, and other tissues. There is evidence that patients taking mycophenolate mofetil frequently experience diarrhea [24], which is associated with duodenal villous atrophy and erosive ileitis [25,26]. It may contribute to vitamin B12 malabsorption. Vitamin B12 deficiency may promote CVD [27,28], which is an important cause of death in kidney transplant patients [29].

The aim of our study was to determine the impact of the rs602662 (G>A) polymorphism of the *FUT2* gene on the functionality of transplanted kidneys and the risk of CVD in transplant patients.

## 2. Results

### 2.1. Characteristics of Patients Qualified for the Study

Among the patients who qualified for the study, there were more men than women (57.5% vs. 42.5%), and their ages ranged from 19 to 82 years. Patients under 50 years of age constituted 38.06% of the group, most people, 55.72%, were between 51 and 70 years of age, and only 6.22% of people were over 70 years of age. The mean age for the group taking CsA was 54.02 ± 12.60 and was statistically significantly higher than in the second group: 51.00 ± 13.37 (*p* = 0.021). Analyzing the age of patients, it was observed that cyclosporine was more often taken by the oldest patients ≥70 years of age (8.20% vs. 4.56%), while the youngest <40 years of age took tacrolimus (24.66% vs. 13.12%). No statistically significant differences were observed comparing the mean height and blood pressure, both systolic and diastolic. The body weight of patients with CsA was higher at 78.77 ± 17.06 kg vs. 75.52 ± 15.35 kg in the TAC group (*p* = 0.045). The mean BMI values did not differ statistically significantly between the groups and were 27.12 ± 5.47 kg/m^2^ and 26.25 ± 4.35 kg/m^2^ in the CsA and TAC groups, respectively (*p* = 0.082). Patients taking cyclosporine were more likely to be underweight (1.64% vs. 0.91%) and obese (27.87% vs. 15.98%), while those taking tacrolimus were more likely to be of normal weight or overweight. Comparing the time since transplantation in both study groups, it was observed that in the CsA group, the average time was 122.40 ± 76.51 months (min–max: 1–324), and in the TAC group 76.41 ± 52.43 months (min–max: 1–264), with *p* < 0.001.

Chronic diseases were frequently reported in patients after kidney transplantation, most often hypertension and diabetes, but their incidence was not statistically different in both study groups. However, more chronic comorbidities were observed in the CsA group compared to the TAC group (21.31% vs. 13.24%, *p* = 0.044). There were no statistically significant differences in the use of stimulants (alcohol and cigarette smoking) between the groups. Analyzing the self-assessment of their health condition, 15.42% of patients answered that they felt very good, 72.64% good, 11.69% poor, and one person (0.25%) described their health as very poor. The self-assessment of cyclosporine patients was not statistically different from tacrolimus patients (*p* = 0.806). Detailed characteristics of renal transplant patients treated with cyclosporine and tacrolimus are presented in Table 1.

The patients underwent laboratory blood tests (morphology, renal profile, liver function tests, and lipid profile). The obtained results are presented in Table 2. Analyzing the morphology of patients after kidney transplantation, a higher number of white blood cells (WBC), red blood cells (erythrocytes, RBC), and an increase in hematocrit (HCT) and hemoglobin (HGB) concentration were noted in patients taking TAC compared to CsA group. The differences for RBC and HGB were statistically significant between the analyzed groups (RBC—4.24 ± 0.59 vs. 4.42 ± 0.61, with *p* = 0.004; 37.99 ± 4.77 vs. 39.11 ± 5.14, with *p* = 0.026). However, the number of thrombocytes (PLT, platelets) was higher in the CsA group, and the median was 215.50 (174.00–258.00) vs. 197.00 (158.50–240.00) in the TAC group, with *p* = 0.004. Analysis of the renal profile showed a higher median urea nitrogen concentration in patients taking preparations containing cyclosporine at 26.45 mg/dL (20.60–35.40) vs. 22.95 mg/dL (17.60–33.30), with *p* = 0.004. Upon analyzing liver parameters, no differences were observed in the concentrations of alanine aminotransferase (ALAT) and aspartate aminotransferase (ASPAT), while the CsA group had a statistically significantly higher median bilirubin of 0.54 mg/dL (0.43–0.71) vs. 0.44 mg/dL (0.33–0.62) in the TAC group, with *p* < 0.001. When assessing the lipid profile, no significant differences were observed between patients taking cyclosporine and tacrolimus (Table 2).

### 2.2. Analysis of the FUT2 rs602662 Variant

In the group of 402 tested patients, 33% had the homozygous GG genotype, 49% had the heterozygous GA genotype, and 18% had the homozygous AA genotype. In the next stage of the study, the frequencies of genotypes and alleles of the *FUT2* rs602662 polymorphism were compared in a group of 183 patients with CsA and 219 patients with TAC. The statistical analysis performed using the chi-square test showed that the genotype frequencies for this variant were consistent with the Hardy–Weinberg equilibrium (HWE) law. The observed frequency of rs602662 alleles of the *FUT2* gene was similar in the analyzed groups. The A allele was present in 43.7% of patients taking cyclosporine and in 41.1% of those taking tacrolimus (OR = 0.898; 95% CI: 0.678–1.189; *p* = 0.453).

Table 3 shows the genotype frequency of *FUT2* rs602662 polymorphism in renal transplant patients treated with cyclosporine and tacrolimus. In the group treated with cyclosporine, the GG genotype was present in 32.2% of the subjects, the GA in 48.1%, and the AA in 19.7%. A similar distribution was obtained in the TAC group: GG—33.8%, GA—50.2%, and AA—16.0%. There were no statistically significant differences in any of the analyzed genetic models between the analyzed patient groups. Also, after adjusting the regression model for age, BMI, and time since transplantation, no statistically significant differences were observed. In both cases, the recessive genetic model was shown to be the best. For the raw regression model, the OR = 0.78; the 95% CI: 0.46–1.30; *p* = 0.335; and the AIC = 557.1, and the adjusted OR = 0.74; the 95% CI: 0.44–1.25; *p* = 0.260; and the AIC = 552.3.

### 2.3. Associations of Genotypes and Alleles of the FUT2 Gene with Clinical and Biochemical Data

**Figure 1:**
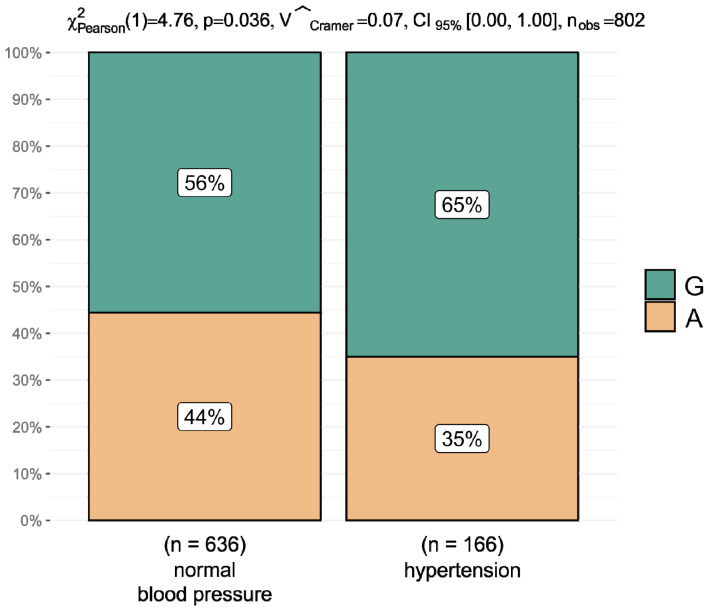
Percentage distribution of rs602662 alleles of the *FUT2* gene and arterial hypertension in the entire group of 402 patients after kidney transplantation.

A comparative analysis of the mean clinical data depending on the genotypes and alleles of the rs602662 variant was performed in renal transplant patients treated with cyclosporine and tacrolimus. Upon analyzing the data for the entire group of 402 patients, an association of genotypes containing the G allele with a higher incidence of hypertension was observed. Hypertension occurred in 25.00% of patients with the GG genotype, in 21.21% of those with heterozygotes, and in 11.27% of patients with the AA genotype. However, the AA genotype was present in 88.73% of patients without hypertension, the AG in 78.79%, and the GG in 75.00% (*p* = 0.068). Statistically significant relationships were obtained by comparing the frequency of hypertension and alleles of the rs602662 variant. The G allele was present in 65% of people with hypertension and in 56% of patients with normal blood pressure (*p* = 0.036) (Figure 1).

In terms of age, weight, height, BMI, blood pressure, and gender, no statistically significant differences were observed between genotypes in the group of patients taking cyclosporine. In the TAC group, differences in the mean age and height of patients were shown to be statistically significant (Table 4). Then, the results of the laboratory tests were analyzed depending on the genotypes in groups of patients after kidney transplantation treated with cyclosporine and tacrolimus.

In 183 patients taking cyclosporine, the most interesting observation was the difference in the alanine transaminase (ALAT) medians. In patients with the GG genotype, the highest values were noted at 19.00 U/L (IQR: 13.00–30.50), they were lower in heterozygous patients at 15.50 U/L (IQR: 11.00–21.00), and they were the lowest in AA homozygotes at 15.00 U/L (IQR: 12.00–19.00), with *p* = 0.081 (Table 5). Comparing the concentrations of this transaminase for alleles of the rs602662 variant, the median for the G allele was statistically significantly higher—16.00 U/L (IQR: 12.00–27.00)—compared to the median for the A allele—15.00 U/L (IQR: 12, 00–20.00), with *p* = 0.029.

In patients taking tacrolimus, a statistically significant difference was noted in the medians of aspartate transaminase (ASPAT). In patients with the GG genotype, the lowest value was 16.00 U/L (IQR: 14.00–21.00), and the highest value in patients with the heterozygous genotype was 19.50 U/L (IQR: 16.00–25.00), with *p* = 0.008 (Table 6).

## 3. Discussion

In patients after kidney transplantation, special attention is paid to avoiding the risk of side effects and comorbidities that may cause transplant failure or death, in particular obesity, infections, cancer, and cardiovascular diseases. Vitamin B12 deficiency may contribute to the occurrence of CVD, which is one of the main causes of death in patients after kidney transplantation. In our study, we determined the impact of the rs602662 (G>A) polymorphism of the *FUT2* gene on the functionality of transplanted kidneys and the risk of CVD in transplant patients.

Upon analyzing data for the entire group of 402 patients, we observed an association of genotypes containing the G allele with a higher incidence of hypertension. Hypertension occurred in 25.00% of people with the GG genotype, in 21.21% of those with heterozygotes, and in 11.27% of people with the AA genotype. However, the AA genotype was present in 88.73% of patients without hypertension, the AG in 78.79%, and the GG in 75.00% (*p* = 0.068). Statistically significant relationships were obtained by comparing the incidence of hypertension and alleles of the rs602662 variant. The G allele was present in 65% of people with hypertension and in 56% of people with normal blood pressure (*p* = 0.036). The lipid profile in relation to the tested genotypes was comparable in patients taking cyclosporine or tacrolimus.

As shown, the *FUT2* gene is highly polymorphic and shows variable frequency in populations [30]. Hazra et al. performed a meta-analysis to identify the loci associated with plasma vitamin B12, homocysteine, folic acid, and vitamin B6 [31]. They demonstrated an association of vitamin B12 in plasma with rs602662 and rs492602 polymorphisms in strong linkage disequilibrium (LD) with rs601338 polymorphism and the *FUT2* W143X mutation. They confirmed that carriers homozygous for non-secretory variants had higher vitamin B12 levels than carriers of the secretory genotype [31]. Another study showed that white women who had the GG genotype for the rs492602 polymorphism of the *FUT2* gene, which is strongly associated with *FUT2* rs601338 polymorphism, had higher plasma vitamin B12 concentrations [15]. A meta-analysis conducted in Italy (Tuscany and Sardinia) and the USA (Baltimore, Washington) showed that the presence of the A allele for the rs6022662 polymorphism was associated with a higher concentration of vitamin B12 [32].

This relationship confirms that our patients with the AA genotype have a lower risk of hypertension compared to the G allele and GG genotype of the rs6022662 polymorphism. This is the first study in a European population assessing the impact of the rs6022662 polymorphism of the *FUT2* gene on the occurrence of CVD in patients after kidney transplantation. Our study indicates that patients with the G allele and GG genotype have an increased risk of hypertension, which may lead to CVD. This is because vitamin B12 deficiency is strongly associated with hyperhomocysteinemia, which is a major risk factor for CVD.

In addition, the evaluation of the renal parameters showed no effect of the *FUT2* polymorphism on the risk of organ rejection, because the levels of creatinine, eGFR, potassium, and urea nitrogen were prognostic of successful transplantation.

## 4. Materials and Methods

### 4.1. Patients

The study included 402 patients after kidney transplantation treated with immunosuppression who were divided into two groups taking cyclosporine (CsA, n = 183) and tacrolimus (TAC, n = 219). The patients were recruited at the Division of Nephrology and Kidney Transplantation, the Independent Public Provincial Hospital in Szczecin, and the Department of General Surgery and Transplantation, Pomeranian Medical University in Szczecin.

After renal transplantation, immunosuppressive therapy consisting of a calcineurin inhibitor (TAC/CsA), mycophenolate mofetil, and glucocorticoids is the standard treatment. Underage patients, retransplant patients, and patients with rapidly progressive graft failure, severe neurocognitive disease, or inability to communicate fluently in Polish were not eligible for the study.

Peripheral blood was used for biochemical tests (renal, hepatic, and lipid profile parameters), the measurement of drug blood concentrations (cyclosporine, tacrolimus), and the analysis of the genetic variants. Fasting whole blood concentrations of TAC and CsA weredetermined before drug administration. The analysis was performed using the ARCHITECT i2000SR analyzer (Abbott, Chicago, IL, USA). The ARCHITECT System Tacrolimus was used to determine drug concentrations based on chemiluminescent microparticle immunoassay (CMIA) according to the manufacturer’s protocol. Clinical and biochemical parameters were evaluated to determine the risk for graft rejection.

### 4.2. Analysis of the FUT2 rs602662 (G>A) Polymorphism

Genetic analysis was carried out at the Department of Stem Cells and Regenerative Medicine at the Institute of Natural Fibers in Poznan, Poland. Genomic DNA was isolated from peripheral blood leukocytes using a commercial kit—Macherey-Nagel NucleoSpin^®^Blood (Macherey-Nagel GmbH&Co, Düren, Germany)—according to the manufacturer’s protocol. The analysis of the FUT2 rs602662 (G>A) polymorphism was performed using real-time PCR using LightCycler^®^96 (Roche Diagnostics, Rotkreuz, Switzerland). A set of LightSNiP rs602662 for *FUT2* polymorphism contained appropriate concentrations of specific primers and probes for the amplified fragment and was prepared according to the manufacturer’s instructions. The PCR program was initiated at 95 °C for 10 min. Each PCR cycle comprised a denaturation step at 95 °C for 10 s, an annealing step at 60 °C for 10 s, and an elongation step at 72 °C for 15 s (45 cycles). The final stage was the melting of products as a result of temperature rise to 95 °C. The analysis of the genotyping was based on the melting curve using LightCycler^®^96 Basic Software (16 February 2023). The research was approved by the Bioethics Committee of Poznan University of Medical Sciences, Poland (no. 510/12;574/18), and all patients gave written informed consent to participate before enrolling in the study.

### 4.3. Statistical Analysis

The R program version 4.2.2, the “SNPassoc” version 2.0-2 and the “ggstatsplot” package were used for statistical analyses of the results [33,34,35]. The normality of data distribution on the interval scale was assessed using the Shapiro–Wilk test. Normally distributed variables were presented as arithmetic means with standard deviation (SD), and non-parametric variables were presented as medians with lower and upper quartiles (Q1; Q3). Assessing the difference in the compared parameters between groups, when a normal distribution was obtained, the Student’s *t*-test or analysis of variance (ANOVA) and Tukey’s post hoc test were used. Interval data without a normal distribution and data on an ordinal scale were assessed using the Mann–Whitney or Kruskal–Wallis test. To compare data on a nominal scale presented as numbers and percentages, χ^2^ or Fisher tests were used, thus depending on the size of the compared groups. Correlations were assessed using Spearman’s rho coefficient for non-parametric data.

The comparison of allele frequencies in groups with the expected frequencies resulting from the Hardy–Weinberg equation was made using the χ^2^ test. Unconditional logistic regression was used to calculate odds ratios (ORs) with 95% confidence intervals to demonstrate the association of the rs602662 polymorphism of the *FUT2* gene in kidney transplant patients treated with immunosuppression divided according to the calcineurin inhibitor taken. A crude model and one adjusted for factors with significant differences between groups (age, BMI, and time since transplantation) were included, and odds ratios were calculated to compare allele and genotype frequencies. Five genetic models were analyzed (codominant, dominant, recessive, overdominant, and log-additive). The Akaike information criterion (AIC) was chosen to evaluate the model. Results with *p* < 0.05 were considered statistically significant.

## 5. Conclusions

In conclusion, our results suggest that the *FUT2* rs6022662 polymorphism may influence the risk of CVD. This is because it has been shown that patients with the G allele and the GG genotype have an increased risk of hypertension, which may lead to CVD. Upon analyzing renal parameters, it was demonstrated that there was no effect of *FUT2* polymorphism on the risk of organ rejection, because the levels of creatinine, eGFR, potassium, and urea nitrogen were prognostic of successful transplantation.

The advantages of our study should also be mentioned. First, a relatively large group of post-kidney transplant patients with detailed clinical data was collected. Second, this is the first study to evaluate the effect of the *FUT2* polymorphism on CVD risk in kidney transplant patients. The limitation of the study is that homocysteine and vitamin B12 levels were not measured to further confirm the conclusions. This is due to the fact that the data collected so far were intended to assess the impact of polymorphisms involved in the metabolism of immunosuppressive drugs.

## Figures and Tables

**Table 1:** General characteristics and clinical data of renal transplant patients treated with cyclosporine and tacrolimus.

Parameter	Cyclosporine N = 183	Tacrolimus N = 219	*p*
Gender, n (%)			χ^2^ = 0.225 df = 1 *p* = 0.635
women	75 (40.98%)	96 (43.84%)	
men	108 (59.02%)	123 (56.16%)	
Age (years), average ± SD	54.02 ± 12.60	51.00 ± 13.37	0.021
Age, n (%)			χ^2^ = 13.327 df = 4 *p* = 0.010
<40	24 (13.12)	54 (24.66)	
40–49	36 (19.67)	39 (17.81)	
50–59	61 (33.33)	51 (23.29)	
60–69	47 (25.68)	65 (29.68)	
≥70	15 (8.20)	10 (4.56)	
Blood pressure (mmHg), average ± SD			
systolic	132.60 ± 13.20	131.93 ± 11.85	0.593
diastolic	82.81 ± 8.35	82.22 ± 7.62	0.461
Body weight (kg), average ± SD	78.77 ± 17.06	75.52 ± 15.35	0.045
Height (cm), average ± SD	169.75 ± 9.49	169.32 ± 9.47	0.652
BMI (kg/m^2^), average ± SD	27.12 ± 5.47	26.25 ± 4.35	0.082
BMI, n (%)			χ^2^ = 9.939 df = 3 *p* = 0.019
<18.5 underweight	3 (1.64)	2 (0.91)	
18.5–24.99 normal weight	68 (37.16)	86 (39.27)	
25–29.99 overweight	61 (33.33)	96 (43.84)	
≥30 obesity	51 (27.87)	35 (15.98)	
Time since transplantation (months), median (IQR)	120 (60–180)	72 (36–108)	<0.001
Time since transplantation (years), n (%)			χ^2^ = 44.854 df = 4 *p* < 0.001
<1 year	11(6.01)	17 (7.76)	
1–5 years	34 (18.58)	65 (29.68)	
5–10 years	41 (22.40)	88 (40.18)	
10–20 years	85 (46.45)	48 (21.92)	
>20 years	12 (6.56)	1 (0.46)	
Chronic diseases, n (%)			
hypertension	41 (22.40)	42 (19.27)	0.516
diabetes	32 (17.49)	50 (22.94)	0.221
other	39 (21.31)	29 (13.24%)	0.044
Use of stimulants, n (%)			
alcohol	40 (21.98)	40 (18.26)	0.423
cigarettes	27 (14.84)	39 (17.81)	0.507
Self-assessment of health status, n (%)			χ^2^ = 0.980 df = 3 *p* = 0.806
very good	27 (14.75)	35 (15.98)	
good	134 (73.22)	158 (72.15)	
poor	22 (12.02)	25 (11.42)	
very poor	0 (0.00)	1 (0.46)	

BMI—body mass index.

**Table 2:** Characteristics of laboratory data of renal transplant patients treated with cyclosporine and tacrolimus.

Parameter	Cyclosporine N = 183	Tacrolimus N = 219	*p*
WBC (10^9^/L)	7.40 (6.02–9.38)	7.85 (6.36–9.56)	0.068
RBC (10^12^/L)	4.24 ± 0.59	4.42 ± 0.61	0.004
HGB (g/dL)	12.86 ± 1.69	13.22 ± 1.86	0.050
HCT (%)	37.99 ± 4.77	39.11 ± 5.14	0.026
PLT (10^9^/L)	215.50 (174.00–258.00)	197.00 (158.50–240.00)	0.004
Na (mmol/L)	141.00 (140.00–143.00)	141.00 (139.00–143.00)	0.501
K (mmol/L)	4.17 (3.86–4.49)	4.13 (3.84–4.38)	0.267
Urea nitrogen (mg/dL)	26.45 (20.60–35.40)	22.95 (17.60–33.30)	0.004
Creatinine (mg/dL)	1.44 (1.19–1.88)	1.44 (1.15–1.84)	0.631
eGFR (mL/min/1.73 m^2^), n (%)			χ^2^ = 2.067 df = 2 *p* = 0.356
<30 (G1 and G2)	25 (13.66)	35 (15.98)	
30–59 (G3a and G3b)	110 (60.11)	116 (52.97)	
>60 (G4 and G5)	48 (26.23)	68 (31.05)	
Uric acid (mg/dL)	7.00 (5.90–8.00)	6.60 (5.90–7.70)	0.101
Bilirubin (mg/dL)	0.54 (0.43–0.71)	0.44 (0.33–0.62)	<0.001
ALAT (U/L)	16.00 (12.00–23.00)	17.00 (12.00–24.00)	0.445
ASPAT (U/L)	18.00 (16.00–23.00)	18.00 (15.00–22.50)	0.167
Total cholesterol (mg/dL)	185.00 (160.00–212.50)	182.50 (162.50–206.00)	0.852
HDL (mg/dL)	59.85 (47.65–75.65)	59.35 (45.85–70.10)	0.492
LDL (mg/dL)	91.00 (69.50–115.50)	97.00 (78.00–117.50)	0.319
TG (mg/dL)	126.00 (93.00–171.00)	134.50 (98.00–177.00)	0.524
Total lipids(mg/dL)	616.00(542.00–707.00)	630.50 (561.00–710.00)	0.376

WBC—white blood cells, RBC—red blood cells (erythrocytes), HCT—hematocrit HGB—hemoglobin, PLT—platelets, Na—sodium, K—potassium, eGFR categories according to KDGIO (Kidney Disease, Improving Global Outcomes), ALAT—alanine transaminase, ASPAT—aspartate transaminase, eGFR—estimated glomerular filtration rate, TG—triglycerides.

**Table 3:** Frequency of rs602662 genotypes of the *FUT2* gene in patients after kidney transplantation treated with cyclosporine and tacrolimus.

Genotype/Model	Cyclosporine N = 183 (%)	Tacrolimus N = 219 (%)	Crude Model			Model Adjusted for Age and BMI		
			OR (95%CI)	*p*	AIC	OR (95%CI)	*p*	AIC
GG	59 (32.2)	74 (33.8)	1.00	0.628	559.1	1.00	0.530	554.3
GA	88 (48.1)	110 (50.2)	1.00 (0.64–1.55)			0.99 (0.63–1.54)		
AA	36 (19.7)	35 (16.0)	0.78 (0.44–1.38)			0.73 (0.41–1.32)		
Dominant	124 (67.8)	145 (66.2)	0.93 (0.61–1.42)	0.742	558.0	0.91 (0.60–1.39)	0.677	553.4
Recessive	147 (80.3)	184 (84.0)	0.78 (0.46–1.30)	0.335	557.1	0.74 (0.44–1.25)	0.260	552.3
Overdominant	95 (51.9)	109 (49.8)	1.09 (0.74–1.61)	0.669	557.9	1.10 (0.74–1.63)	0.644	553.4
Log-additive	183 (45.5)	219 (54.5)	0.90 (0.68–1.19)	0.452	557.5	0.88 (0.66–1.17)	0.368	552.8

BMI—body mass index.

**Table 4:** General characteristics and clinical data of patients after kidney transplantation treated with cyclosporine and tacrolimus depending on the genotypes of the *FUT2* rs602662 variant.

Parameter	Cyclosporine N = 183				Tacrolimus N = 219			
	GG (N = 59)	GA (N = 88)	AA (N = 36)	*p*	GG (N = 74)	GA (N = 110)	AA (N = 35)	*p*
Gender, n (%)				0.588				0.731
Women	21 (35.59%)	38 (43.18%)	16 (44.44%)		30 (40.54%)	51 (46.36%)	15 (42.86%)	
Men	38 (64.41%)	50 (56.82%)	20 (55.56%)		44 (59.46%)	59 (53.64%)	20 (57.14%)	
Age (year), mean ± SD	56.34 ± 10.25	52.38 ± 13.03	54.22 ± 14.62	0.174	50.61 ± 14.34	52.95 ± 12.10	45.66 ± 13.88	0.018
Body weight (kg), mean ± SD	82.19 ± 18.92	77.35 ± 15.26	76.54 ± 17.43	0.165	75.08 ± 15.80	74.64 ± 14.82	79.17 ± 15.93	0.303
Height (cm), mean ± SD	170.46 ± 9.84	169.55 ± 9.53	169.08 ± 8.99	0.763	170.82 ± 10.01	167.75 ± 9.39	171.09 ± 7.78	0.046
BMI (kg/m^2^), mean ± SD	28.11 ± 5.07	26.90 ± 4.81	26.71 ± 5.71	0.286	25.59 ± 4.15	26.47 ± 4.44	26.92 ± 4.42	0.242
Blood pressure (mmHg), mean ± SD								
systolic	134.24 ± 14.01	132.44 ± 13.50	130.28 ± 10.82	0.363	131.01 ± 11.29	133.26 ± 12.92	129.71 ± 8.99	0.220
diastolic	82.63 ± 7.21	82.95 ± 8.66	82.78 ± 9.44	0.973	82.03 ± 7.63	82.29 ± 7.98	82.43 ± 6.57	0.959

BMI—body mass index.

**Table 5:** Characteristics of laboratory data depending on the genotypes of the *FUT2* rs602662 variant in patients after kidney transplantation treated with CsA.

Parameter	Cyclosporine (N = 183)			
	GG (N = 59)	GA (N = 88)	AA (N = 36)	*p*
WBC (10^9^/L)	7.96 (6.25–9.77)	7.32 (6.09–9.37)	6.84 (5.75–9.12)	0.618
RBC (10^12^/L)	4.33 ± 0.50	4.20 ± 0.62	4.23 ± 0.62	0.386
HGB (g/dL)	13.23 ± 1.58	12.64 ± 1.69	12.80 ± 1.81	0.113
HCT (%)	38.97 ± 4.38	37.43 ± 4.88	37.81 ± 4.99	0.158
PLT (10^9^/L)	212.50 (179.00–258.00)	220.00 (175.50–259.00)	213.00 (165.00–245.00)	0.753
Na (mmol/L)	142.00 (140.00–143.00)	141.00 (140.00–143.00)	141.50 (140.00–142.00)	0.827
K (mmol/L)	4.20 (3.96–4.49)	4.16 (3.79–4.50)	4.17 (3.96–4.48)	0.836
Urea nitrogen (mg/dL)	24.60 (19.90–31.60)	27.65 (21.60–37.35)	26.40 (20.70–40.45)	0.186
Creatinine (mg/dL)	1.39 (1.19–1.71)	1.62 (1.20–1.99)	1.40 (1.27–1.87)	0.170
eGFR (mL/min/1.73 m^2^), n (%)				0.166
<30 (G1 and G2)	3 (5.08%)	16 (18.18%)	6 (16.67%)	
30–59 (G3a and G3b)	37 (62.71%)	53 (60.23%)	20 (55.56%)	
>60 (G4 and G5)	19 (32.20%)	19 (21.59%)	10 (27.78%)	
Uric acid (mg/dL)	7.00 (6.20–8.00)	7.10 (5.90–8.05)	6.65 (5.75–7.55)	0.503
Bilirubin (mg/dL)	0.56 (0.40–0.68)	0.52 (0.41–0.68)	0.53 (0.44–0.80)	0.608
ALAT (U/L)	19.00 (13.00–30.50)	15.50 (11.00–21.00)	15.00 (12.00–19.00)	0.081
ASPAT (U/L)	19.00 (16.00–23.50)	18.00 (14.50–23.00)	18.00 (16.00–22.50)	0.478
Total cholesterol (mg/dL)	187.00 (169.50–214.50)	179.00 (156.00–211.00)	186.00 (171.50–213.50)	0.359
HDL (mg/dL)	61.00 (48.65–74.10)	60.60 (47.20–75.40)	58.60 (49.55–72.45)	0.971
LDL (mg/dL)	95.00 (69.50–117.00)	84.00 (73.00–114.00)	98.00 (67.50–115.00)	0.723
TG (mg/dL)	127.00 (111.50–166.00)	123.00 (88.00–168.00)	129.50 (86.50–197.50)	0.805
Total lipids (mg/dL)	622.00 (579.00–677.50)	587.00 (528.00–722.00)	640.00 (551.00–721.50)	0.522

WBC—white blood cells, RBC—red blood cells (erythrocytes), HCT—hematocrit HGB—hemoglobin, PLT—platelets, Na—sodium, K—potassium, ALAT—alanine transaminase, ASPAT—aspartate transaminase, eGFR—estimated glomerular filtration rate, TG—triglycerides.

**Table 6:** Characteristics of laboratory data depending on the genotypes of the *FUT2* rs602662 variant in patients after kidney transplantation treated with TAC.

Parameter	Tacrolimus (N = 219)			
	GG (N = 74)	GA (N = 110)	AA (N = 35)	*p*
WBC (10^9^/L)	8.12 (6.94–9.59)	7.68 (6.20–9.30)	8.26 (6.50–10.49)	0.156
RBC (10^12^/L)	4.46 ± 0.65	4.38 ± 0.61	4.47 ± 0.55	0.586
HGB (g/dL)	13.38 ± 2.04	13.12 ± 1.75	13.18 ± 1.84	0.656
HCT (%)	39.51 ± 5.44	38.93 ± 5.07	38.83 ± 4.78	0.713
PLT (10^9^/L)	197.00 (162.00–231.00)	189.00 (149.00–236.50)	207.00 (163.50–252.00)	0.308
Na (mmol/L)	141.00 (140.00–143.00)	141.00 (139.00–143.00)	141.00 (140.00–143.50)	0.480
K (mmol/L)	4.09 (3.84–4.36)	4.15 (3.86–4.35)	4.04 (3.76–4.56)	0.876
Urea nitrogen (mg/dL)	22.00 (16.40–30.00)	25.60 (18.10–36.30)	21.10 (18.20–29.50)	0.284
Creatinine (mg/dL)	1.46 (1.16–1.81)	1.44 (1.15–1.96)	1.40 (1.12–1.79)	0.894
eGFR (mL/min/1.73 m^2^), n (%)				0.948
<30 (G1 and G2)	12 (16.22%)	18 (16.36%)	5 (14.29%)	
30–59 (G3a and G3b)	40 (54.05%)	59 (53.64%)	17 (48.57%)	
>60 (G4 and G5)	22 (29.73%)	33 (30.00%)	13 (37.14%)	
Uric acid (mg/dL)	6.60 (5.70–7.70)	6.70 (5.90–7.60)	6.70 (5.90–7.80)	0.844
Bilirubin (mg/dL)	0.43 (0.33–0.63)	0.44 (0.32–0.61)	0.46 (0.35–0.64)	0.916
ALAT (U/L)	16.00 (12.00–22.00)	18.00 (13.00–25.00)	17.00 (12.00–21.00)	0.120
ASPAT (U/L)	16.00 (14.00–21.00)	19.50 (16.00–25.00)	17.00 (13.50–20.00)	0.008
Total cholesterol (mg/dL)	183.00 (168.00–205.00)	185.00 (161.00–209.00)	180.00 (161.50–202.50)	0.551
HDL (mg/dL)	58.60 (44.90–66.90)	61.20 (50.25–74.10)	59.45 (42.80–76.00)	0.446
LDL (mg/dL)	98.50 (78.00–119.00)	100.00 (79.00–117.50)	88.50 (75.00–102.00)	0.268
TG (mg/dL)	121.00 (95.00–173.00)	139.50 (101.50–180.50)	119.50 (103.00–186.00)	0.739
Total lipids (mg/dL)	614.50 (551.00–709.00)	636.00 (570.00–723.00)	626.00 (541.00–686.00)	0.567

ALAT—alanine transaminase, ASPAT—aspartate transaminase, eGFR—estimated glomerular filtration rate.

## Data Availability

Data are contained within the article.
